# Medical and Physical *Intelligence*

**Published:** 1799-04

**Authors:** 


### MEDICAL AND PHYSICAL

### *INTELLIGENCE,*

### (Original and Selected.)

A Work of the firft rank has juft made its appearance in Germany, whether we confider the importance of the fubject, the celebrity of the two authors, whofe obfervations are detailed in it, or the real excellence of its interefting contents. It is intitled, "*A Treatife on the Faculty of the Mind to conquer the Senfations produced by Difeafes, by means of the fimple Act of the Will.*" This is the production of the venerable philofopher Kant; it is publifhed by Hufeland, and accompanied with notes by that refpectable phyfician; fo that we have before us the joint labours of two great men, on the moft ufeful of all arts, that of *prolonging life*. So valuable and interefting a treatife demands more particular notice, and we fhall endeavour to give the fubftance of it in the next Number of this Journal.

---

Profeffor Tode of Copenhagen, has lately publifhed, at Leipzig, an *Analyfis* of a German book, intitled, "*Preliminaries of a Medical Peace, or Points of Union between* Brown *and his Opponents,*" which analyfis, he prefaces with the following fevere remarks.

"I merely announce this book, without determining its price. I am more than ever convinced, that we ought not to lay hold of, or examine, an apple of difcord thrown upon the ground. I have always thought, that the Brunonian fyftem contains feveral truths which are not new, and feveral novelties that are not true. We have no right to blame thofe who demand proofs in confirmation of Brown's affertion, illuftrated by cafes of patients, and who pay no attention to theories unfupported by experience. It is undoubtedly difgraceful to the fchool of medicine in Germany, that fuch an author, of very little eftimation in his own country, fhould actually produce a revolution, and that fo many medical men think that they have hitherto been enveloped in complete ignorance, until it pleafed Providence to raife up this precious inftrument, to reveal medical truths. I, too, once imagined, that I had difcovered the path of truth, but the young people, who ought certainly to have fome underftanding, raifed fuch loud clamours, that I found it neceffary to remain filent. The fyftem of Brown, in the mean time, has opened an inexhauftible mine to the book-makers. To what a multiplicity of *explanations*, *commentaries*, *critiques*, and *apologies*, has it given birth; and behold, at length, the *preliminaries of peace*, not concluded by plenipotentiaries, but conceived in the head of an anonymous dreamer! Inftead of examining the Brunonian fyftem (and above all, where he pretends to have made a difcovery of a truth), inftead of citing the real difcoverer, who had formerly laid down what Brown takes advantage of, inftead of criticifing rigoroufly the great number of falfe precepts which originate with Brown himfelf, the author appears to believe, and perhaps does believe, that the fancies of this vifionary are applicable to practice; and hence, inftead of peace, we fhall only have *Brunonianifm corrected and purified.*"

The

The female Citizen Adrienne Liquiere, a pupil of the *Anti-Cæſarean School* of Citizen Sacombe, at Paris, defended on the 19th of December laſt, a public theſis on the mechaniſm of child-birth, for which ſhe was honoured with a prize-medal. Her theſis is divided into five ſections, in which the fair author, with much acuteneſs and ability, endeavours, 1. To explain the *three* ſituations of the child in the uterus, and to prove that the third of theſe ſituations conſtitutes what is called *inſenſible labour;* the phenomenon which takes place during inſenſible labour, being a pathognomic ſymptom of the laſt ſtage of pregnancy: 2. To demonſtrate *geometrically*, that the mechaniſm of child-birth conſiſts in the ſpiral, rotary motion of the body of the child on its axis, at the moment when it clears its narrow paſſage, and the cavity of the ſmall pelvis:—3. To determine the delivery by the head, the feet, the knees, and the buttocks; and to prove that the *thouſand and one* poſitions, which the Cæſarean accoucheurs abſurdly aſſign to the child in the uterus, ought to be reduced to *four;* the delivery by the knees or buttocks being merely modifications of that by the feet:—4. To ſupport her doctrine againſt all objections that may ariſe againſt it:—and 5. To prove that her learned friend, Profeſſor Sacombe, was the firſt diſcoverer of the ſpiral, rotary motion of the child in the uterus; and that two modern authors, the elder Baudelocque and Hamilton, who have availed themſelves of this diſcovery, are only plagiariſts.

---

It is a fact already known, that *urine* contains phoſphorus and ſeveral ſaline combinations with the phoſphoric acid. Citizens Fourcroy and Vauquelin have lately diſcovered, in this liquid, the baſe of alum and phoſphat of magneſia. They obſerved, that a particular animal matter, which characteriſes urine, and from which it acquires all its properties, quickly forms ammoniac; whence it ought to be claſſed among the triple ſalts; it being, in that ſtate, conſiderably leſs ſoluble than before, and capable of being precipitated in a lamellated form, or cryſtalline needles. Theſe two chemiſts have aſcertained the different alterations of which this liquid is ſuſceptible; they have enumerated the ſpontaneous changes to which it is ſubject, and ſhewn that the inveſtigation which they have only begun, is an object deſervingly engaging the attention of phyſicians; inaſmuch, as it may be the means of ſolving one of the moſt important problems in the animal economy of man, both in a healthy and diſeaſed ſtate.

---

Mr. Tennant formerly announced, that the *arthritic concretions* analyſed by him, were a combinaton of the lithic acid and ſoda. This remarkable fact has been confirmed by a recent experiment made by Citizens Fourcroy and Vauquelin. They lately received from Citizen Veau, of Launay, a phyſician of Tours, one of the concretions that ſpontaneouſly ſeparated from a gouty tumour of the fingers of a man whoſe joints were much deformed by the diſeaſe, and his fingers ſwelled to an enormous ſize. They ſubmitted this concrete ſubſtance to chemical analyſis, and found it to conſiſt of the lithat of ſoda, now called the *urat of ſoda,* mixed with a conſiderable quantity of animal matter.

---

The obſervations of Citizen Baume, relative to the decompoſition of the *calcareous muriate of lime,* as connected with the inquiries into the nature of a malady, called by phyſicians the *black,* or *atrabiliary diſeaſe,* of which

Cit.

Cit. PORTAL has developed the causes and effects, by pointing out the remedies to be employed in the cure of it—have engaged the attention of the Class of Physical Sciences, in the National Institute of France. We hope, in a future number, to be enabled to furnish our readers with the result of this interesting inquiry.

---

FOURCROY has recently published, in the '*Annales de Chimie*,' a Memoir containing his observations on the experiments made by the celebrated English chemist MAYOW, who about a century ago discovered many chemical truths, that bear a very striking resemblance to those lately demonstrated by the French chemists. The works of this philosopher, says Fourcroy, were first brought into general notice by the edition of them published by Dr. BEDDOES, in 1790. Of this learned 'Memoir,' we propose to give a more particular account in our next number. Let it suffice, for the present, to remark, that although Fourcroy does great justice to Mayow, whose merits and talents he admits to be greater than those of his cotemporaries, yet he is of opinion, that Mayow did not sufficiently pursue and demonstrate the principles of which he was the author; and that, in fact, he was not fully sensible of the importance of these discoveries, far less of the extent to which they might be carried. In short, Mayow only conjectured what the conclusive results, together with the luminous and scientific doctrines of modern chemistry, have reduced to mathematical certainty.

---

In the Italian Annals of Chemistry we meet with a letter from Dr. CARRADORI to Citizen DUPRE, expressing some doubts as to the solidity of the New System of Chemistry. The Doctor declares that he cannot yet admit the *ensemble* of the new doctrine, but only some evident facts, or points demonstrated. He thinks that the combination of oxygen, during combustion, can hardly act a principal part in this operation, but must be accessory and foreign; the base of this gas having only a proper tendency (independent of the affinity or action which determines the combustion) to unite itself to bodies deprived of a portion of their phlogistic, carbonic, or any other principle of inflammability, by a considerable elevation of temperature. This combination might be made in a manner similar to that of water with alkali and caustic lime.

Neither has the composition of water, according to Carradori, been satisfactorily proved, on which the whole edifice of LAVOISIER principally depends: the inflammable air obtained, is furnished by the body which serves to decompose the water, and not by that liquid. "The Italians will always object," says he, "that you *suppose* what requires to be *demonstrated*." If electricity decomposes water, why should not fire also decompose it? Nor is the experiment of the recomposition of water any more approved of by this author. The residuum, which he supposes always to remain after the condensation of the two gases, gives room to object, that all water was dissolved in airs, and only adhered to the elastic state, by the aëriform residuum, and some other incoercible element.

The author might adduce other facts, such as those which demonstrate the existence of an inflammable principle in certain combustible bodies, for example, sulphur; but, to use his own words, "*he declines these means of defence, having arms more than sufficient to combat the French errors.*"

Elements coercible, which give to water the form and nature of as many different gases as we are acquainted with; an *inflammable principle*, carbon, hydrogen, or phlogiston, which is a matter of no consequence, and which quits combuftible fubftances according to a temperature fomewhat elevated; thefe, fay the French chemifts in reply, are the well-proved things which Dr. Carradori puts in place of the moft notorious facts, on which the ftructure of the new chemiftry is founded.

---

The French National Inftitute have paid particular attention to the learned foreigners fent to Paris by their allies, to affift in the operations relative to *a general uniformity of weights and meafures*. They are admitted to the private fittings of the inftitute, as well as to its library, and each of them has lately received elegant prefents, particularly a highly embellifhed copy of Didot's magnificent edition of Virgil. One of thefe foreign literary characters, however, Mr. Bruggs, from Denmark, has unfortunately given much offence; for it is alledged, that in his correfpondence with Copenhagen, he has faid every thing derogatory to the dignity of the inftitute, and reprefented it in very odious colours.

---

Brugnatelli has juft publifhed a difcourfe on *Light*, which he confiders in *three different modifications*; namely, light chemically combined; light fimply aggregated, but in a latent ftate; and light fenfibly aggregated. The firft of thefe is not to be feparated from a body, but by the effect of elective attraction. Oxygenous gas, phofphorus, fulphur, &c. contain it in that ftate.

---

It appears, from a number of experiments, made with great accuracy by Citizen Brugnatelli, on the means of producing detonatory fulminations, by phofphorus combined with different bodies, that, 1. The nitrate of filver, both cryftallized and melted, fulminates when ftruck with phofphorus, even at a low temperature, and that all the falts of filver do not fulminate in the fame manner, nor with the fame degree of violence; 2. That the greateft number of metallic nitrates fulminate with phofphorus: 3. That common nitre detonates with phofphorus alone, although the fulminating property of nitre was known only by mixing it with feveral combuftible bodies, and elevating it to a certain temperature, or by placing it into contact with an inflamed body, as gunpowder, or other fulminating powders; 4. That thofe falts which contain no nitre in their compofition, are not fulminating; 5. That the oxygenated muriates of pot-afh, filver, and mercury, fulminate with phofphorus; but the two laft more feebly than many other falts; 6. That falts are not the only bodies which fulminate with phofphorus, fome metallic oxyds poffeffing the fame property; 7. That phofporus is likewife not the only acidifiable and folid combuftible body capable of producing fulminations, charcoal being attended with a fimilar effect, at a more elevated temperature; and 8. That the bodies fulminating with phofphorus produce no fuch phenomenon when plunged into the oxygenated muriatic acid in a liquid ftate; neither do they fulminate, when expofed, jointly with phofphorus, to a high temperature. The ftroke of the hammer is neceffary to put in ofcillation the component parts of bodies, in order to determine their affinities with a fufficient degree of accuracy. Thefe inferences are deduced from the author's experiments, fourteen in number, which he concludes with an obfervation, that the oxygenated muriate of pot-afh, is well-known to produce effects fuperior to nitre mixed with charcoal and fulphur, and converted into gunpowder; and that it detonates by percuffion or trituration, with a great number of combuftible fubftances; but that he did not expect effects much more confiderable in ufing fimple nitrates, and even metallic calces mingled with phofphorus, and ftruck with a hammer.

The

The uncommon degree of cold, which prevailed at Paris on a particular day of laſt winter, has given Citizens Vauquelin and Fourcroy an opportunity of repeating the experiments formerly made by Prof. Lowitz of Peterſburg, for producing artificial cold: the *decimal thermometer* ſtood, on that day, at 17°, or 13° and ſix tenths of Réaumur. When this new thermometer was at 70° below the freezing point, they mixed eight parts of the muriatè of lime, and ſix parts of ſnow looſely gathered. This mixture immediately generated an incalculable degree of cold: 20 pounds of quickſilver were completely frozen into a ſolid maſs in half a minute, as well as ſpirit of wine, ether, the baſis of vinegar, &c. On dipping the tip of the finger into this mixture, it loſt all ſenſe of feeling within four minutes, became as white as paper, and did not recover its ſenſation till held for ſome time in the mouth. At the firſt moment of immerſion, an acute pain was felt in the finger, as if violently compreſſed in a vice. This artificial cold may be eſtimated at 40 degrees.

---

Two poſthumous volumes of intereſting chemical tracts, by the celebrated Pierre Bayen*, have juſt been publiſhed at Paris, by his nephew, Citizen Malatret; of which we intend, in a future number, to give a detailed account. In the mean time, we ſhall mention only that, among other valuable diſſertations theſe tracts conrain anylyſis of the mineral waters of Luchon, accompanied with a number of chemical experiments; a pictureſque deſcription of the valley and ſprings of Luchon; an examination of the ſaline efflorefcenſes found near the mineral ſources of the ancient Roman baths; an analyſis of the mercurial ſyrup of Belet; an inveſtigaton of a mine of ſphatic iron (minera ferri alba ſpathi formis); and an analytical account of different ſtones and marbles;—all which ſubjects are treated in a manner worthy of the great reputation of the deceaſed author.

---

In the 83d Number of the "*Annales de Chimie*," we find a memoir by Citizen Proust, on the chemical combinations of *tin*, which the author has publiſhed as a ſupplement to Pelletier's inveſtigations, relative to the different degrees of oxygenation of which that metal is ſuſceptible in a ſtate of ſolution by acids.

Citizen Prouſt agrees with Bergman's obſervations, that tin oxydated *ad minimum*, acquires only thirty parts in the hundred, and ſtill contains ſome portion of the muriatic acid. If made red hot in the crucible, it parts with the muriate of tin in a gaſeous form; and when the tin is oxydated *ad maximum*, it acquires forty parts in the hundred, but it may be eaſily reduced to thirty; and then it is of a bluiſh colour, and inſoluble in acids. The author concludes his memoir by ſaying, that although Pelletier made no mention of the muriate of white copper, he is fully perſuaded, that this chemiſt was acquainted with the fact, from his having paſſed over the deſoxydation of that metal by tin, in a manner which ſhewed his intention to reſume the ſubject in a future occaſion. But, while he does every juſtice to Pelletier, he affirms, in a manner carrying conviction, that although his operations coincided with thoſe of his friend, they were originally contrived by himſelf.

---

* Bayen, late member of the National Institute in the Section of Chemisty, was born in 1725, at Chalons, in the department of Marne. He died after a painful and lingering illness, which he bore with uncommon fortitude for twelve years, in the month of Pluviose, in the sixth year of the French Republic (January, 1798) at the advanced age of 72.—The French have lost in him a virtuous Citizen, and an excellent practical Chemist. W.

In the National Inftitute of France, Citizen LHERETIER has prefented the clafs of phyfical fcience with two new fpecies of plants; the one difcovered at Madagafcar by Citizen BRUGUIERE in the courfe of his voyage round the world, and which the Inftitute has termed *Bruguiera,* in memory of their affociate; the other, difcovered by the fame learned naturalift in the Ifle of France, belongs to the family of orchis, and to which Lheretier propofes to give the name of *Rhizodendrum.* It is a fpecies of acacia, originally a native of North America, and is called by Vauquelin *Robinia Vifcofa,* from the glutinous matter which gathers on its furface. This fubftance much refembles that of refins properly fo called, excepting that it is with difficulty foluble in alcohol, particularly when cold. Ether is its true folvent, and the refult is a folution of pale green. In feparating the ether when cold, the matter is extracted which forms the epidermis of the Robinia; it has neither flavour nor fenfible fmell; is extremely glutinous; is eafily foftened by the heat of the fingers, to which it clofely adheres; fwells when burnt, and leaves behind a portion of coal. It readily unites with fat and oil, but not with alkalis, and hardly with alkohol; on being expofed to the air, it does not dry like common refins. On account of thefe properties, Citizen Vauquelin confiders this as a new vegetable production, which affords an unufual proportion of refinous matter.

---

A new work of the firft confequence to the naturalift and botanift, will foon be publifhed at Leipzig—the fplendid *Flora Americana Septentrionalis,* by C. I. HUTTER, of Philadelphia, who avails himfelf of this great *Book-fair,* to introduce to the learned of Europe this joint production of fcience and art. The drawings are made from nature, and a number of finifhed plates are already executed by the celebrated artift Eckftein, formerly Painter and Engraver to the Duke of Mecklenburgh. The colouring of the plates is intrufted to no other but profeffional botanifts. Upon the whole, this work will unqueftionably be the chief production of art, from the weftern Continent. Mr. Hunter announces in the Jena Gazette, that he hopes to be able to tranfmit the firft numbers to Germany early in the fpring. They will contain either non-defcripts, or fuch plants as have hitherto been but imperfectly defcribed.

---

*Citizen* DESFONTAINES has fupplied an important defideratum in the fcience of Botany, by the complete *Flora of Mount Atlas,* a work of uncommon merit, which has very lately made its appearance at Paris.

---

*Citizen* BROUSSONET, whofe zeal for fcience has led him to travel into various parts of Africa, has juft publifhed an accurate account of the procefs employed, at Fez and Tetuan, for preparing goat-fkins, in making Morocco of different colours; together with correct defcriptions of the plants ufed in Barbary for manufacturing this valuable article.

---

Mr. SNEYD, of Belmont in Staffordfhire, has communicated to the Society for the encouragement of the Arts, &c. *a method of preferving the feeds of plants, in a ftate fit for vegetation,* by keeping or fending them furrounded either by raifins or brown moift fugar; which, upon a comparative trial, he found much preferable to packing them in abforbent paper; in the former cafe, the feeds were found frefh and healthy, while in the latter, there was a prevailing drynefs, and they were infefted by infects.

---

The ingenious Mr. FORSYTH, his Majefty's gardener at Kenfington, has lately difcovered, and liberally communicated to the public, an effectual

*remedy for deftroying an infect*, which has within thefe few years appeared in the *Britifh orchards*, and threatens deftruction to the apple-trees. Thefe minute and imperceptible infects are lodged, in millions, in white efflorefcences formed upon the trees, and not unlike thofe which may fometimes be feen on ftones in the field. The following is Mr. Forfyth's compofition, which has the additional merit, that it is cheap and eafily procured. To any optional quantity of human urine, add as much cow-dung and lime as will reduce the whole to the confiftence of common paint: with this mixture the infected trees muft be anointed about the latter end of March; and this fimple precaution will fully anfwer the defired effect.

---

Citizen LAMARCK, in a memoir refpecting the *new claffification of fhells*, lately read before the National Inftitute of France, has judicioufly pointed out the neceffity of increafing the number of genera, and more accurately afcertaining the characters which diftinguifh thefe fanciful productions of nature. He extends their number to one hundred and feventeen, whereas Linnæus and other naturalifts have never exceeded fixty. This improvement of Lamarck will greatly facilitate the acquifition of fcientific knowledge, in that amufing branch of natural hiftory.

---

A gentleman of Long-Ifland, in America, has lately difcovered there a new variety of iron-ore of the argillaceous kind. Walking on his farm, and obferving the various mineral productions under his feet, he took notice of a fmall fpot which had the appearance of being paved with ftones of a regular figure. On taking up fome of them, he found their ftructure the fame with the common argillaceous iron-ftone which lay fcattered about in rude lumps, but their fhape was nearly pentagonal. They were about four inches long, and ftood perpendicular, clofe to each other, in a ftiff loam: in fome, four fides only were to be traced. The number of thefe ftones was thirty-fix, evidently formed from a folid circular mafs of argillaceous iron-ore of about two feet five inches in breadth, and four inches in thicknefs in the central parts, and becoming thinner towards the edges. In the natural fracture of the ftone, the pieces had feparated in thefe regular forms, much refembling bafaltes; and like bafaltes, though figured, they were not cryftallifed, but as completely terrigenous and opaque as any argillaceous iron-ore. Thefe fpecimens go very far towards deciding the difpute, if any doubt ftill remain, of the igneous or aqueous origin of bafaltes. They fupport by the moft powerful evidence the latter doctrine; as they cannot be fuppofed to be a volcanic production—the American gentleman agreeing in opinion with BERTMAN, WEIDMAN, and KIRWAN, that moft, if not all bafaltic columns, have been formed by cryftallization.

---

A committee of the *Medical Society of New-York*, confifting of Meffrs. TILLERY, RODGERS, and MITCHILL, were chofen at a meeting of that body, lately held for the exprefs purpofe of inveftigating the *origin*, *caufe*, *and prevention of the peftilential diftempers*, which for fome years paft have fo terribly afflicted New-York, and other cities and towns of the United States. We hope, the report of the committee will fhortly appear; and as they have been promifed the aid of the Municipality, as well as of the Health office, their means of procuring information will be greatly facilitated, and thus an opportunity offered of laying before their fellow-citizens a much wanted ftatement of facts, to elucidate the true nature of the yellow fever.

---

There is at prefent a confiderable controverfy at Paris, on the fubject of *Inoculation for the fmall-pox*. From the great mortality which lately prevailed.

prevailed, when that disorder was contagious, several writers endeavour to persuade government to prohibit inoculation, at least within the precincts of towns. The advocates for inoculation, on the other hand, contend, that such an inderdiction would be similar to the *arretés* of the old Parliament, against emetics, and indeed against inoculation itself—arretés which so much disgraced France. They deny that the contagion has become more frequent since the practice of inoculation, while the disease itself has thus been rendered less virulent and fatal; and they add, that were inoculation to become universal, it would be the means of extirpating the disorder, as no person would remain liable to infection.

In the mean time, the *cow-pox*, which has of late been the subject of much discussion in this country, has also attracted the attention of the French physicians. In the *Decade Philosophique* of the 10th Ventôse (20th February) we meet with a particular account of Dr. JENNER's treatise on this subject. The French reviewer confines himself to an analysis of Dr. Jenner's work, and does not attempt to give a conclusive opinion on the question now at issue before the public. He however justly remarks, that if the inoculation for the cow-pox prevent the infection of the small-pox, the author who promotes so beneficial a practice, instead of being reprobated for the propagation of of a *new* contagious malady, renders an essential service to mankind.

---

Dr. BRERA, Professor of the Practice of Medicine in the University of Pavia, lately announced a work intitled, '*Ratio medendi, qua in clinico instituto Ticinensi, ab initio mensis Decembris*, 1796, *ad finem usque Junii*, 1797, *usus est*, &c. Having, however, considered, that were this work to appear in the Latin language only, its practical utility would be much confined, the Professor has judiciously determined to publish a translation from the original manuscript in the vernacular language of Italy.

In this work, the author proposes particularly to place in a more conspicuous light the *Brunonian Theory of Medicine*; to inquire into its merits: to refute, by actual observation, those points which rest upon erroneous principles, and to illustrate others which have a practical tendency. The whole work is to be divided into two parts, each of which will form one folio volume, embellished with four plates, by the celebrated engraver Anderloni.

Of this splendid work Messrs. ORELL, FUESSLI, and Co. at Zurich, have advertised a German translation, to be conducted under the immediate superintendance of the author, who has likewise agreed to furnish them with impressions from the original plates. We trust that this country will not be long without a translation of so valuable a work.

---

Among other translations of useful Medical books, from the English into the German language, Dr. MICHAELIS, of Leipzig, has lately announced a correct version of Dr. GEORGE FORDYCE's original and classical work, intitled, "*A Third Dissertation on Fever*, Part I. containing the history and method of treatment of a regular continued Fever." The two former Dissertations, by the same author, and likewise translated by Dr. Michaelis, were received in Germany with universal approbation.

---

The *Physical Journal*, formerly conducted by the celebrated Professor *Gren*, who died of a nervous fever, on the 26th of November last, at Halle, in his 39th year, will be continued under the new title of *Annals of Physics*, by Professor GILBERT, of the same University, who has undertaken the office of editor. Two numbers of this excellent work have already appeared, and and Mr. Gilbert hopes to be enabled to furnifh a number every month, but, the particular time for its publication is not fpecified.

The fecond volume of the "*Syftem of Pharmacology*," begun by the late Profeffor *Gren*, will be completed from the materials found after his demife, by two of his learned friends, who, by a public advertifement in the Literary Gazette of Jena, have pledged themfelves to publifh this volume in October next.

---

Dr. Koelner, a clergyman of Gotha, has lately publifhed a German tranflation of the "*Chemico-phyfiological Effays*," originally written in Latin, by *J. Mayow*, in 1681. This tranflation is embellifhed with fix engravings, and accompanied with a learned hiftorico-critical preface, from the pen of Dr. *Scherer*, Counfellor of mines to the Duke of Saxe-Weimar. Dr. Scherer alfo propofes fhortly to publifh his own Commentary on the works of Mayow.

---

Mr. Achard, of Berlin, has made feveral experiments, with a view to determine the *effects* of *electricity* on the fermentation of vegetable fubftances, and the corruption of dead animal bodies. From the refult of his inquiries it clearly follows, that electricity is a remarkable agent in thefe proceffes of nature, as it has an obvious tendency to accelerate both; and that the fudden putrefaction of flefh, whether raw or dreffed, muft be afcribed folely to the more abundant accumulation of electric matter, at that time. Mr. Achard has alfo difcovered, by experiments, that in a body of atmofpheric air, confined in a Leyden flafk, and electrified as ftrongly as poffible, neither abforption nor dilation had take place; and that the fame was the cafe when he expofed the jar to abundance of fparks: from which it appears, that the quality of the air is not changed by electricity, or that the electric matter muft pafs through the interftices of the air, without caufing any feparation of its component parts.

---

The Abbe Spallanzani has lately difcovered a fpring of frefh water, which rifes through the falt water, in the Mediterranean fea, at the diftance of fixty-five feet from the fhore, about a mile from Spezzia. It is about twenty feet in diameter, and thirty-eight and an half in depth. The water at the furface is not frefh, but fomewhat lefs falt than that by which it is furrounded. By means of a medicine invented for the purpofe. Spallanzani afcertained that at the bottom it is perfectly frefh, but exceedingly torbid and flimy. This phenomenon the Abbé confiders to proceed from two fmall ftreams, which flow down a hill in the neighbourhood of Spezzia; and, after being united, throw themfelves into an unfathomable abyfs, from which the water forces its way through the earth, and furnifhes nourifhment to the fountain that rifes in the fea.—Since writing this article, we learn that the world has loft this eminent philofopher.

---

In the new tranfactions of tbe Royal Society of Goettingen, we meet with fome curious and interefting obfervations and experiments, made by Profeffor Beckman, *on ftaining wood*. A piece of plain tree, put into pounded dragons-blood, mixed with oil of turpentine, and placed over the fire, in a glafs veffel, acquired in a fhort time a beautiful mahogany colour. The wood, when ftained, can be eafily freed from the dragons-blood adhering to it, by means of rectified fpirit of wine.—Gamboge, diffolved in fpirit of turpentine, gave the furface of a fmall piece of wood a fhining golden yellow colour, except the fibres and veins, which inclined rather to red. A piece of pear-tree in the fame folution acquired a darker colour, fomewhat approaching ing to green, and in part became nearly of an olive colour: different colours may, therefore, he obtained by uſing different kinds of wood.—One part of dragons-blood, two parts of gamboge, gave to the wood of the plane-tree and beech, a remarkable variety of dyes.

The ſame dyes had nearly a ſimilar effect on the wood, when prepared in ſpirit of wine. When they were merely diſſolved in ſpirit of wine, the extract was not ſufficiently ſtrong; it was neceſſary to boil it over a ſlow fire, till nearly evaporated, but the colour is never ſo bright as that produced by means of an oil. The profeſſor alſo enumerates various experiments with diſſolved ſalts and metals, which have been ſucceſsfully made, but as theſe are already deſcribed by MACQUER, we think it unneceſſary to tranſcribe them.

---

As the ſubject of typhus-contagion, and its hitherto inexplicable effects on the animal frame, are involved in ſo much darkneſs and uncertainty, that it forms, and will perhaps ever continue to form, one of the moſt intereſting problems to the chemiſt and phyſiologiſt, we announce with ſatisfaction a work which, we underſtand, is ready for the preſs, and will be publiſhed without delay; it is is intitled, "*Obſervations deduced from facts and expements, tending to evince the non-exiſtence of typhus-contagion; interſperſed with remarks on animal life, and on thoſe laws by which it is governed:* by JOHN FRANKS, navy ſurgeon, and member of the Corporation of Surgeons of London.

The author is of opinion, that the debilitating effect produced on the animal machine, by the operation of *fear*, occaſioned by the influence which this inviſible, undefined *ſomething* has obtained, is the only contagion which we have to guard againſt; and that every circumſtance of a febrile ſtate may be very ſatisfactorily accounted for, without having recourſe to contagion as a cauſe of diſeaſe. He further obſerves, that beſides the difference of opinion which prevails reſpecting the *nature of contagion*, the evidence of its reality is very defective, and that we are not even poſſeſſed of a correct definition of this inviſible and incoercible agent.

---

Mr. WHITE, a reſpectable practitioner of Mancheſter, is preparing for the preſs a highly intereſting anatomical work which, we underſtand, is in conſiderable forwardneſs, and will ſhortly be publiſhed by DILLY, London. From the accounts we have been able to obtain, this work is chiefly devoted to *comparative anatomy*. It will form one quarto volume, embelliſhed with elegant engravings, and be intitled: *A Treatiſe on the Gradation of Man.*"

---

The *Phyſical Society of Guy's Hoſpital* have propoſed the following prize queſtions, for the year 1799:

*In Medicine:*—What is the origin of the cow-pox, and in what does it differ from the ſmall-pox? Are its effects on the human conſtitution milder than thoſe of the inoculated ſmall-pox—and is a patient, who has been inoculated for the cow-pox, and experienced its conſtitutional effects, equally ſecure from the contagion of the ſmall-pox?

*In Phyſiology:*—How do the vegetable and mineral poiſons act upon the body—and what are the beſt means of preventing their deleterious effects?

Diſſertations in anſwer to the queſtions propoſed, muſt be delivered to the librarian, previous to the firſt Saturday in December, 1799. According to the eſtabliſhed laws of the Society, a motto ſhall be prefixed to each diſſertation, and with it muſt be delivered a ſealed packet, with the ſame motto motto on the outfide, and with the author's name on the infide. Two fets of books, value five guineas each, are annually given to the authors of the two beft differtations on the fubjects propofed.

The two queftions for laft year were as follow: 1, What is the caufe of intermittent fever—and what are the beft means of curing it? 2, What is the proximate caufe of inflammation? We underftand that the fuccefsful candidate, in anfwering both queftions of laft year, was Dr. WARWICK, of Rotherham, Yorkfhire.

---

The medical world has lately fuftained an irreparable lofs by the death of FREDERIC ALBRECHT CHARLES GREN, Profeffor of Medicine in the Univerfity of Halle, and member of feveral learned focieties in different parts of Europe. He was equally eminent as a judicious practitioner in phyfic, as an excellent teacher, and one of the ableft chemifts Germany has given birth to. By a cool and fteady oppofition to the contefted principles of the prefent antiphlogiftic fyftem, the truth of which he, at length, himfelf admitted, he has done infinite fervice to the chemical fcience; while he cultivated Natural Philofophy with an ardour almoft unexampled. The celebrated '*Journal der Phyfick*' has been conducted by him, during the laft ten years, and is now continued by one of his learned friends, Profeffor GILBERT, of Halle, under the title of '*Annales der Phyfick* *.'

Gren is the author of various and valuable publications, the principal of which are the following: '*A fyftematic Manual of Chemiftry*,' firft printed in two volumes octavo, but re-publifhed in a fecond improved, and enlarged edition, confifting of four volumes, about the year 1795:—'*Principles of Pharmacy and the Materia Medica*,' in two volumes;—'*Obfervationes et Elementa circa Genefin aëris fixi et phlogifticati*;'—'*Obfervations on Fermentation and the Projects thence obtained*,' &c.

We lament the early demife of this favourite friend of fcience, whofe indefatigable exertions for the improvement of mankind, and the honour of his country, have been fatally terminated by a nervous fever—the endemic difeafe of the German literati—when he had fcarcely attained his 38th year, and his labours and productions had become objects of admiration, in Germany as well as in France. The chemifts of the latter country never mention his name but with refpect, and the New Syftem of Chemiftry found in him, fucceffively, an acute opponent, and able fupporter. He was born at Bernburg, a fmall town in Germany, on the firft of May, 1760, and died at Halle, univerfally regretted, on 26th of November, 1798.

---

Geneva, the fmall but important republic in the various departments of literature and the fciences, has loft, on the 22d of January laft, one of her moft celebrated and dignified fons, HORACE BENEDICT DESAUSSURE, aged 58, who has long adorned his native city, as an excellent naturalift, chemift and mineralogift.

In his 21ft year, he was appointed Profeffor of Philofophy at the Lyceum of Geneva; an office which he held with much honour to himfelf, and benefit to the ftudents that attended his lectures, till the year 1786, when he refigned it to his former pupil and coadjutor, the prefent Profeffor PICTET, who ably fulfils that difficult tafk, after fucceeding fo learned a man.

Defauffure was the founder of the Society of Arts, to which Geneva is in a great meafure indebted for the great reputation and profperity it has attained, during

---

* Vide page 193 of this number.

during the last thirty years, and of which he continued the president until his death. As a member of the Council of Two Hundred, and afterwards, as a member of the National Assembly of Geneva, he had frequent opportunities of testifying his patriotic zeal. He lately had the honor of being appointed Professor of Natural Philosophy at the National Institute of France; an appointment, however, the active duties of which his impaired state of health did not permit him to discharge. Among other works of this celebrated philosopher, we shall mention only his '*Travels in the Alps*,' in four volumes, octavo; '*Essays on Hygrometry*;' and his '*Observations on the Epidemics of leaves*.'

He has left behind him two sons, and an amiable daughter. With the accomplishments of her sex, she combines an extensive knowledge of the sciences; and his eldest son is already known as a respectable chemist, and adept in Natural Philosophy.

